# Facile sonochemical method for preparation of Cs_2_HgI_4_ nanostructures as a promising visible-light photocatalyst

**DOI:** 10.1016/j.ultsonch.2021.105827

**Published:** 2021-11-12

**Authors:** Elham Abkar, Abbas Al-Nayili, Omid Amiri, Mojgan Ghanbari, Masoud Salavati-Niasari

**Affiliations:** a Institute of Nano Science and Nano Technology, University of Kashan, Kashan P. O. Box. 87317-51167, Islamic Republic of Iran; b Department of Chemistry, College of Education, University of Al-Qadisiyah, Diwaniya 1753, Iraq; c Department of Chemistry, College of Science, University of Raparin, Rania, Kurdistan Region, Iraq

**Keywords:** Cesium mercury tetraiodide, Organic pollutants, Environmental remediation, Sonochemical pathway, Thermochromic materials

## Abstract

Attempts are continuing to discover novel and efficient solutions to promote water grade and industrial sewage treatment. For the first time, we present a novel Cs_2_HgI_4_ photocatalyst functional below visible radiation. Cs_2_HgI_4_ nano photocatalyst has been prepared via an accelerated sonochemical approach to examine its photocatalytic progression. Several construction circumstances, including variations of power and time of sonication and performance of different surfactant types, were conducted to produce fine particles with uniform morphology. FESEM images attested that the presence of surfactant had an adverse and destructive effect on the morphology of products. The bandgap for Cs_2_HgI_4_ nanostructures was determined to be approximately 2.3 eV, making these nanostructures desirable for photocatalytic applications. The photocatalytic data confirmed that Cs_2_HgI_4_ could destroy acidic coloring agents greater than basic ones. The highest photodegradation was observed for methyl orange with 76.8%.

## Highlights

• The low cost and simple sonochemical method used to fabricate Cs_2_HgI_4_ nanostructures.
• Examination of various states on structure, purity, size, and morphology of the product.
• The presence of surfactants negatively affects the morphology and particle size.
• Photocatalytic ability was scrutinized through different acidic and basic coloring agents.
• Degradation of methyl orange about 76.8% utilizing Cs_2_HgI_4_ nanostructures.

## Graphical abstract

**Figure:**
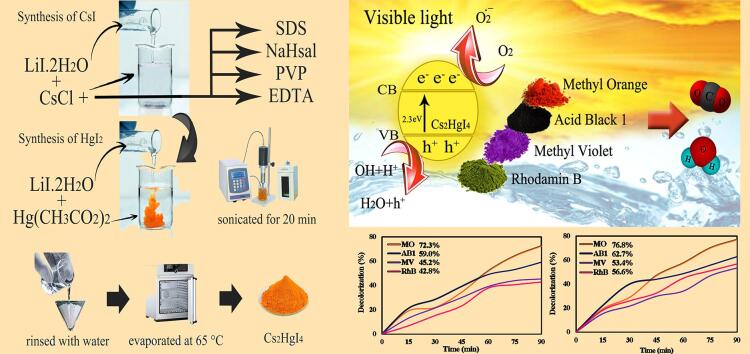

## 1 Introduction

Water is a valuable origin that is vital to each organism around the world. Water covers about 70% of the earth, although just 2.5 % is designated as freshwater. A small quantity of fresh water is utilized, reused, and afterward treated so that it tends to be reused repeatedly [1]. The use of water is not confined to domestic consumption but is also used in a wide range of applications, including agriculture and industrial. According to the UNESCO world water assessment programme (WWAP) in 2003, about 2 million tons of untreated water comes from agricultural and industrial water drainage. Increasing the amount of water consumption increases the amount of sewage. Effluent treatment needs a high cost since the contamination in the sewage must be efficiently eliminated so that water can be safely recycled again. Even with traditional techniques including, physical and biological treatments, more superior technology with shorter time and lower cost is still needed to eliminate pollutants effectively [2]. Therefore, discoveries of the accepted procedures/approaches for wastewater treatment are vital for our current state and maintainable development.

Today, population growth and industrial development not only decrease clean water resources but also worsen water quality, which has a serious impact on the health and safety of living organisms [3]. In addition, wastewater comprises various organic compounds, including dyes, medicines, and personal care products that are often resistant to natural degradation. Although their concentration is very low, the damage cannot be ignored. In the last few years, several procedures have been promoted for the treatment of organic wastewater [4]. Multiple types of materials are used to eliminate pollutants from sewage, such as adsorbents, catalysts (heterogeneous and homogeneous), ozone, membranes of inorganic and organic materials, etc. [5], [6], [7]. The traditional water purification technologies available today, including coagulation or adsorption, do not totally destroy or eliminate the contaminants. In some way, these contaminations were easily being collected by transferring them to a different phase. Therefore, environmentally friendly, highly effective, and efficient purification technologies need to be developed to eliminate organic contaminants from wastewater. Advanced oxidation processes (AOPs) is a newfound sewage processing technique that treats contaminants by producing hydroxyl radicals (•OH) responsible for organic destruction. The hydroxyl radicals oxidize and mineralize nearly every organic molecule due to their strong non-selective oxidation power, producing inorganic ions and CO_2_ as ultimate products [8]. The production of •OH can be started by initial oxidants (ozone, hydrogen peroxide), catalysts (zinc oxide, titania, and Fenton reagent), or energy sources (heat, ultrasonic, and UV light) [9], [10]. Amongst AOPs, photocatalysis is an attractive alternative method that can eliminate the emerging pollutants at ambient pressure and temperature with oxidation.

Cesium mercury tetraiodide, Cs_2_HgI_4_ belongs to an attractive group of M_2_NX_4_ syntheses (N = Hg, Cd, Zn, Mn, Co; X  = Cl, Br, I) comprising isolated tetrahedral NX_4_^2-^ ions [11]. Two groups exist at room temperature in the A_2_BX_4_ compounds either in a monoclinic Sr_2_GeS_4_-type structure with a space group P2_1_/m [12], [13] or an orthorhombic β-K_2_SO_4_-type structure with a space group Pnma [14]. Due to the A_2_BX_4_ compounds containing Cs, practically all of them are crystallized in the orthorhombic β-K_2_SO_4_-type structure [15], [16]; nevertheless, just two syntheses, namely Cs_2_HgI_4_ and Cs_2_CdI_4_, form in the monoclinic structure of Sr_2_GeS_4_-type [17], [18]. However, upon heating, Cs_2_HgI_4_ and Cs_2_CdI_4_ convert from the monoclinic structure of Sr_2_GeS_4_-type (α-phase) to the orthorhombic structure of β-K_2_SO_4_-type (β-phase), and this transmutation is identified as α → β phase transition [19]. For Cs_2_HgI_4_, the α → β phase transition transpires at around 520 K [20] or 523 K [12]. Shimizu et al. [20] showed that α → β transformation in Cs_2_HgI_4_ is a first-order phase transition using Differential Thermal Analysis (DTA).

Environmental pollution has become a global catastrophe these days, so new energy stores, such as fuel cells and solar cells, have become more concentrated. Photodecomposition is one of the most prominent techniques employed in the elimination of contaminants in manufacturing sewage. Scientists desire to utilize available natural sources to gain the power required to decompose organic dyes in industrial effluents. The most relevant natural energy origin is solar radiation that comprises about 47% infrared radiation, 46% visible light, and 5–7% UV light [21], [22]. Photocatalytic oxidation of several toxic organic colorants in manufacturing effluents has been performed on various semiconductor photocatalysts below UV radiation. Research is currently concentrated on obtaining high photocatalytic performance with novel photocatalysts, particularly with solar light. Given the appropriate bandgap of Cs_2_HgI_4_ (2.3 eV), we have decided to consider its photocatalytic ability below visible light for the initial time. In this study, we have employed a sonochemical procedure to compose Cs_2_HgI_4_ nanostructures. Our principal aims are remarked following:

1. Preparation of Cs_2_HgI_4_ nanostructures via a simple and low cost sonochemical route.

2. Study Cs_2_HgI_4_ photocatalytic ability for the initial time as a new and effective photocatalyst for degradation of organic dyes.

## 2 Experimental

### 2.1 Materials

All chemical materials applied were supplied in excellent grade. Cesium chloride (CsCl), Lithium iodide (LiI·2H_2_O), Mercury (II) acetate (Hg(O_2_CCH_3_)_2_), Ethylenediaminetetraacetic acid (EDTA), Polyvinyl pyrrolidone (PVP-25000), Sodium dodecyl sulfate (SDS), Sodium salicylate (NaHSal) 1,4-Benzoquinone (BQ), and benzoic acid (BA) were procured from Merck Company and used without any purification.

### 2.2 Synthesis of Cs_2_HgI_4_

**Scheme 1:**
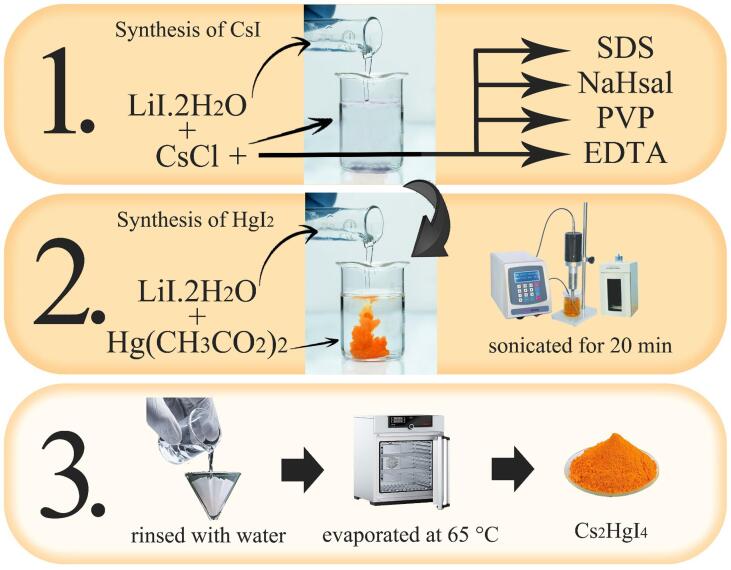
Schematic diagram of the fabrication of Cs_2_HgI_4_ nanostructures by sonochemical method.

Cesium iodide (CsI) was formed through an effortless co-precipitation technique of LiI·2H_2_O (0.27 g, 2.01 mmol) and CsCl (0.34 g, 2.01 mmol). 0.34 g CsCl was dissolved in 15 mL water. A stoichiometric quantity of PVP (0.34 g, 2.01 mmol) was dissolved in 10 mL water and mixed with the CsCl solution. Next, 0.27 g LiI·2H_2_O was dissolved in 10 mL water and mixed with the above solution to obtain CsI. The HgI_2_ was likewise fabricated by combining LiI (0.27 g, 2.01 mmol) with Hg(O_2_CCH_3_)_2_ (0.33 g, 1.005 mmol). The suspension comprising cesium iodide was mixed with HgI_2_ and sonicated for 20 min. The bright orange precipitate was separated, rinsed with water, and eventually evaporated at 65 °C (Scheme 1). The samples in the presence of other surfactants (including SDS, NaHSal, and EDTA) were similarly prepared, and the molar ratio of CsCl to surfactant was 1:1. Table 1 depicts various fabrication circumstances of Cs_2_HgI_4_ for reaching the sought state.

**Table 1:** Preparation conditions for Cs_2_HgI_4._

Sample No.	CsI:HgI_2_ Molar ratio	Type of capping agent	Time of sonication (min)	Power of sonication (W)
1	2	–	20	80
2	2	–	20	60
3	2	–	20	40
4	2	SDS	20	80
5	2	PVP	20	80
6	2	EDTA	20	80
7	2	NaHsal	20	80
8	2	–	10	80
9	2	–	30	80

### 2.3 Photocatalytic ability

Cs_2_HgI_4_ was used in the photocatalytic reaction for photodegradation of several toxic colorants under visible light. A 150 W Osram lamp was exercised as the emission origin, comprising a wavelength between 400 and 780 nm for the photocatalytic procedure. The operations were carried out without light or Cs_2_HgI_4_, and approximately no colorant was degraded after 90 min. 70 mg Cs_2_HgI_4_ was mixed with 50 mL 10 ppm of dye solutions for each operation. The mixture was agitated in the dark for 30 min before activating the light. Every 15 min, A 4 mL example was extracted from the suspension and centrifuged at 8000 rpm for 3 min. The afloat was gathered, filtered, and inspected by a UV–Vis spectrophotometer. The photodegradation percentage (P.D.) was calculated as below [23]:

(1) $$P.D.=\frac{{\text{A}}_{0}-{\text{A}}_{\text{t}}}{{\text{A}}_{0}}\times 100$$

Where A_0_ and A_t_ is the dye absorption before and after light exposure.

## 3 Result and discussion

### 3.1 Characterization

**Fig. 1:**
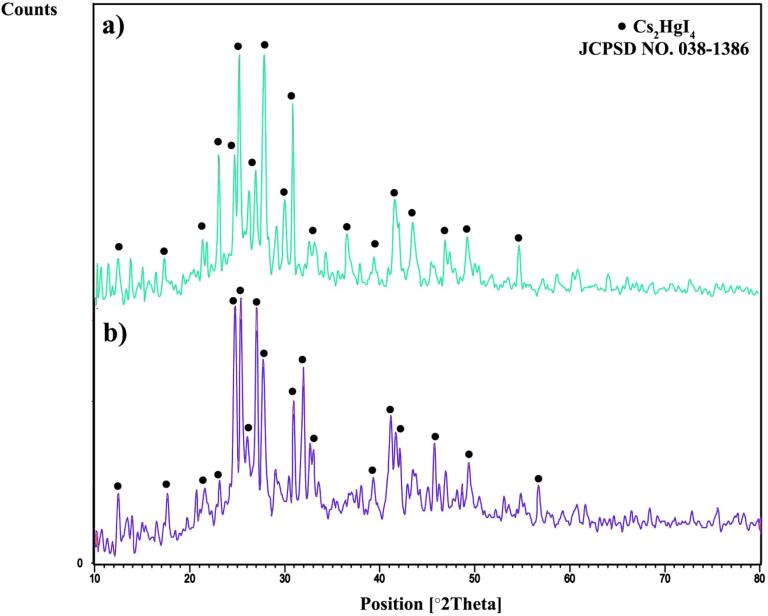
XRD pattern of Cs_2_HgI_4_ in two different power of sonication: a) 80 W (sample 1), and b) 60 W (sample 2).

Fig. 1 reveals the XRD patterns of as-prepared Cs_2_HgI_4_ in two different sonication powers. All diffraction peaks are well-matched with Cesium Mercury Iodide 038–1386. Cs_2_HgI_4_ was formed in a monoclinic structure with a space group of P2_1_/m. Scherrer equation [24], [25] was applied to determine the domain size (D) of about 28.2 and 35.6 nm for samples 1 and 2, respectively.

(2) $$D=\;\frac{K\lambda }{\beta \cos\Theta }$$

Some generic mechanisms are introduced in the fabrication of nanostructures via the sonochemical approach: bubble formation and hot spots, radical generation, and vesicle production [23], [26]. Nevertheless, what is possible in the formation of Cs_2_HgI_4_ nanostructures is the mechanism of the hot spot and bubble formation in the reaction medium. The production and explosion of bubbles raise the temperature, which boosts the reaction toward the product. The reaction progress is synopsized below:

(3) 2CsCl + LiI → 2CsI

(4) Hg(CH_3_COO)_2_ + LiI → HgI_2_

(5) $$2\text{CsI}+{\text{HgI}}_{2}\overset{\text{Ultrasound radiation}}{\to }{\text{Cs}}_{2}{\text{HgI}}_{4}$$

**Fig. 2:**
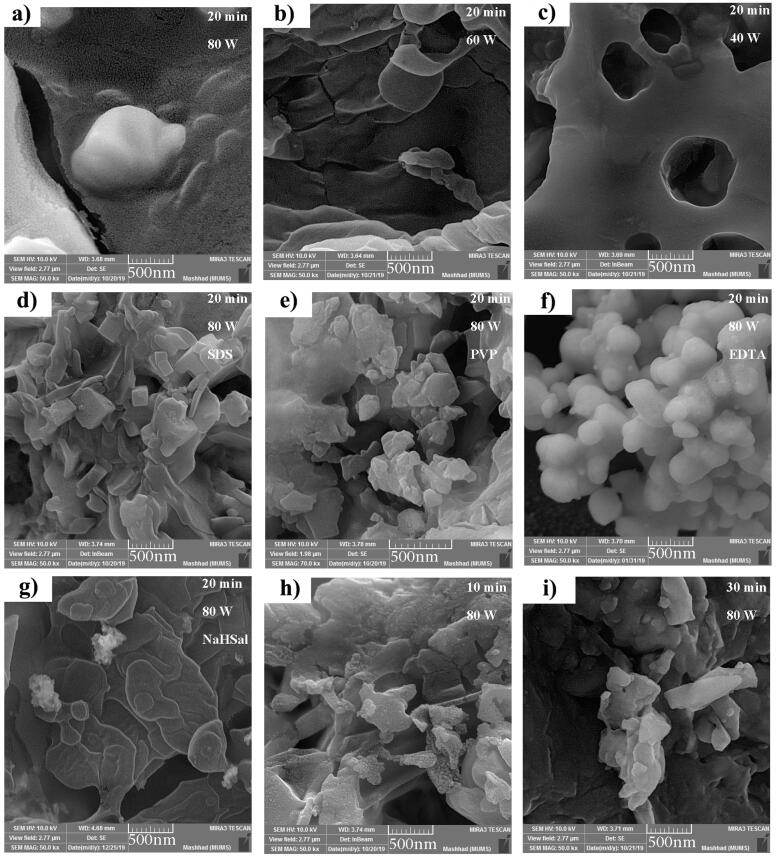
FESEM photographs of as-prepared samples (a) 1, (b) 2 (c) 3, (d) 4, (e) 5, (f) 6, (g) 7, (h) 8, and (i) 9.

SEM photographs were employed to scrutinize the morphology of Cs_2_HgI_4_ nanostructures. Fig. 2(a–c) manifests the FESEM images of Cs_2_HgI_4_ in three sonication powers (80, 60, and 40 W). Increasing the ultrasound power to 80 W has resulted in very fine nanoparticles with an average size of less than 20 nm (Fig. 2a). While reducing the ultrasound power ends up large particles so that at 40 W, a porous micrometer structure with an average pore diameter of 579 nm was formed (Fig. 2c). Thereby, the supreme power was considered at 80 W for 20 min. Fig. 2(d–g) exposes the FESEM photographs of Cs_2_HgI_4_ incorporated with various surfactants, including SDS, PVP, EDTA, and NaHSal, respectively. The resulting photographs explicate that micrometer bulk structures are shaped by using all types of surfactants. Thereupon, surfactants negatively affect the morphology and particle size. Fig. 2h and 2i depict the time effect of sonication on the sample morphology. Reducing the ultrasound time limits the reaction temperature from properly rising, thus creating bulk nanostructures (Fig. 2h). Moreover, a prolonged time (30 min) dramatically raises the reaction temperature, generating misshapen bulky particles (Fig. 2i). As a result, the excellent sonication time was elected at 20 min (Fig. 2a) to arrange uniform Cs_2_HgI_4_ nanostructures (sample 1). The desired sample was applied for the additional examines in the continuation of the study.

**Fig. 3:**
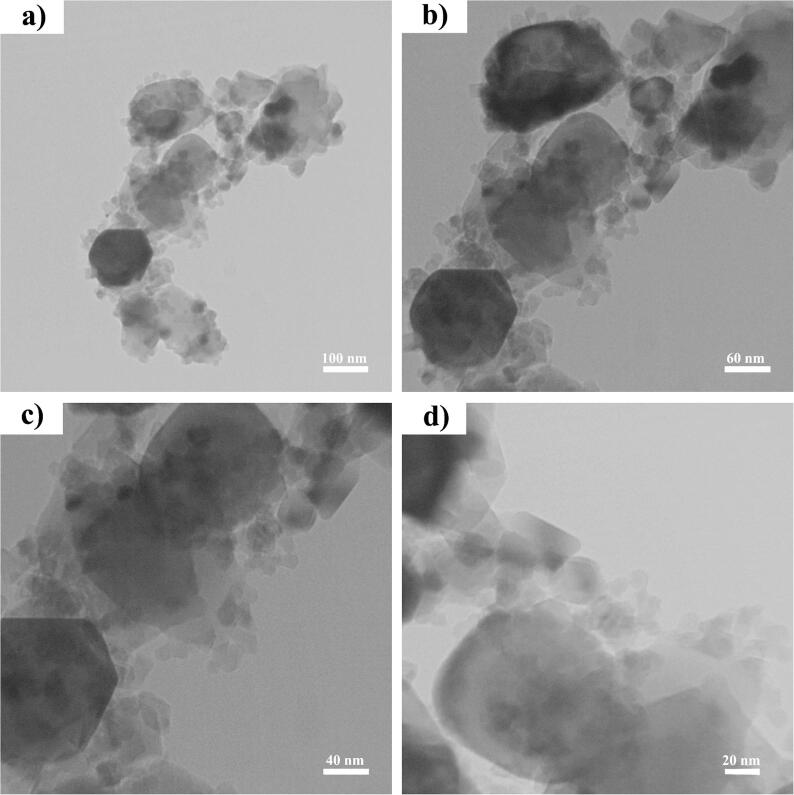
TEM images of sample 1.

Fig. 3 reveals the TEM images of desired sample (sample 1) in multiple scales (20, 40, 60, and 100 nm). Tiny nanoparticles less than 20 nm are formed on large nanoparticles. It was expected in the formation of Cs_2_HgI_4_ nanostructures owing to their high activity.

**Fig. 4:**
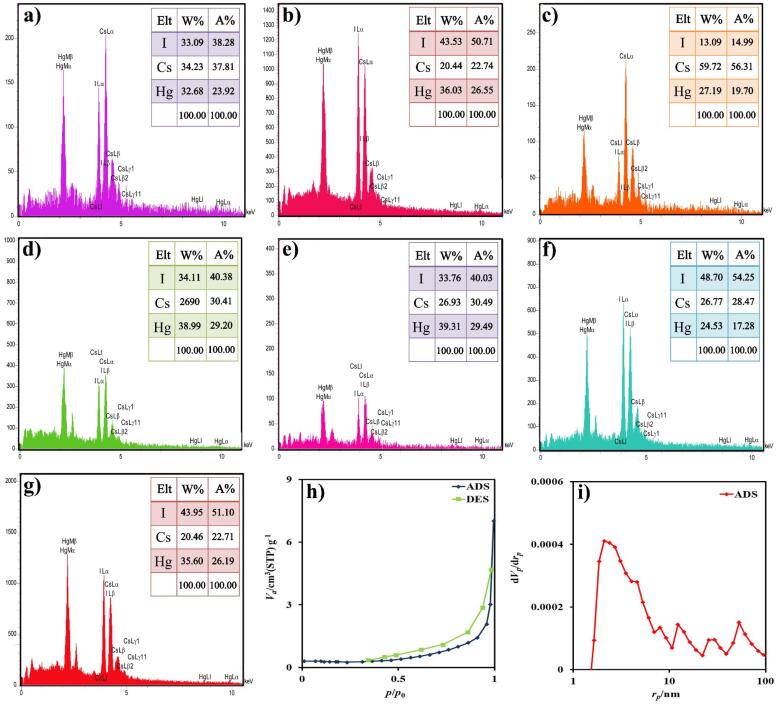
EDS spectrum of the samples (a) 1, (b) 2 (c) 3, (d) 4, (e) 5, (f) 6, (g) 7, (h) N_2_ adsorption/desorption isotherm, and (i) BJH plot of sample 1.

The presence of elements in the Cs_2_HgI_4_ composition was studied by EDS analysis (Fig. 4(a-g)). The EDS outcomes verified I, Cs, and Hg elements in the structure. Additionally, these spectra explicated that there is no element as an impurity in the Cs_2_HgI_4_ compound.

N_2_ adsorption and desorption at 77 K were utilized to estimate the average of pores, total pore volume, and specific surface area of Cs_2_HgI_4_ nanostructures (sample 1). As apparent in Fig. 4h, the type III isotherm was achieved with an H3 loop for this compound [27]. The mean pore diameters, specific surface area, and the total pore volume were calculated at 5.34 nm, 4.283 m^2^g^−1^, and 0.0046 cm^3^g^−1^ employing BET and BJH calculations, respectively. The outcomes confirmed that a suitable surface area of Cs_2_HgI_4_ could be prepared. This surface area notably provides absorption of dyes and the number of active positions in the photo-activated chemical reactions [28].

**Fig. 5:**
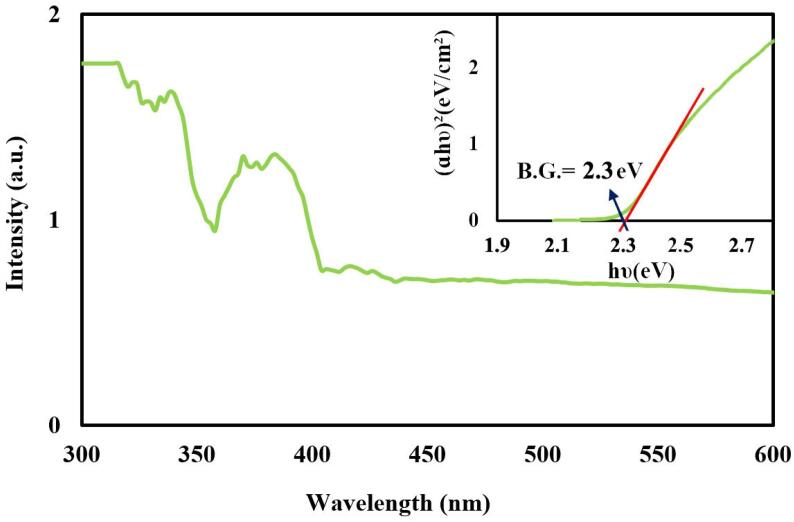
DRS spectrum of sample 1 and inserted diagram to determine the bandgap of Cs_2_HgI_4_ nanostructures.

The bandgap value is one of the principal factors in the identification of semiconductor materials. The bandgap of Cs_2_HgI_4_ was studied by DRS analysis in the wavelength from 300 to 600 nm (Fig. 5). Tauc's formula was applied to predict the bandgap [29]. The conclusions gained from this formula in diagram form of (αhν)^2^ vs. (hν) in the DRS spectrum is explicit. The calculated bandgap for Cs_2_HgI_4_ was estimated at 2.3 eV, which is well-matched to the reported literature [12].

### 3.2 Investigation of photodegradation ability

**Fig. 6:**
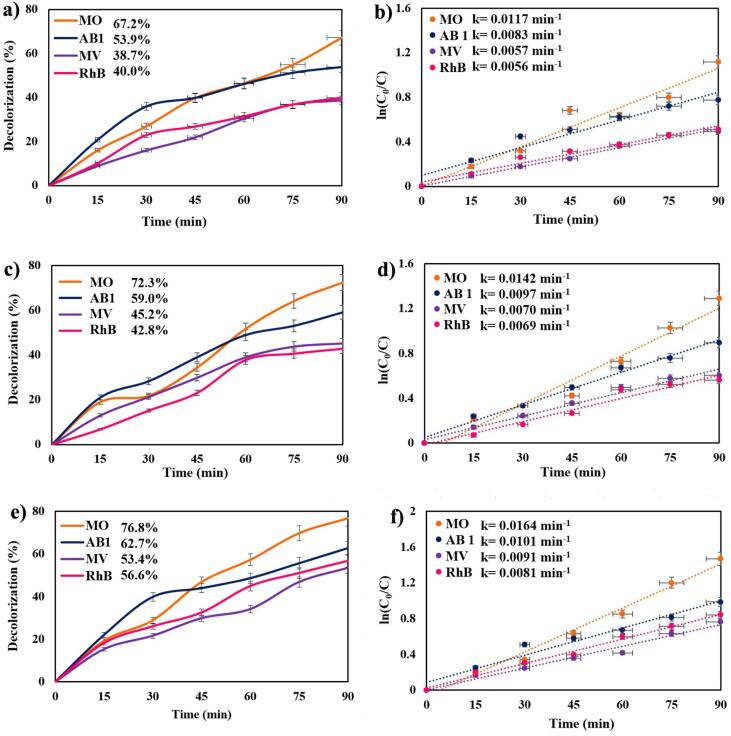
Photodegradation of various organic dyes over sample 1 in three dosages of Cs_2_HgI_4_ a) 0.03 g, c) 0.05 g, and e) 0.07 g, Plots of ln(C_0_/C) vs time b) 0.03 g, d) 0.05 g, and f) 0.07 g below visible radiation.

**Scheme 2:**
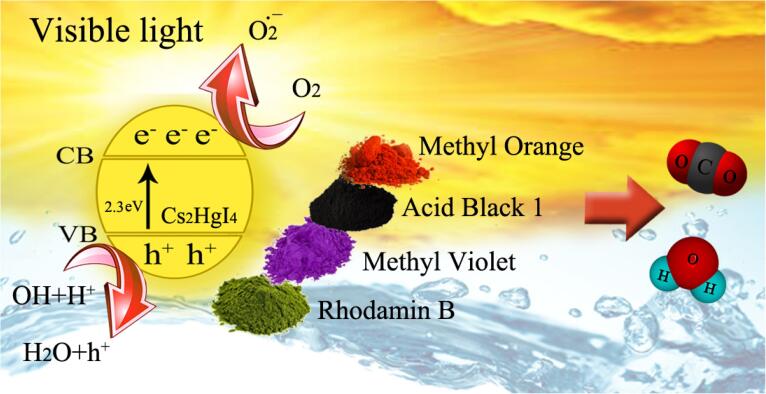
Schematic diagram of the mechanism for the photodegradation of Cs_2_HgI_4_ nanostructures over multiple dyes.

Cs_2_HgI_4_ nanostructures were applied as a photocatalyst to survey the photodegradation over toxic dyes under visible light due to their suitable bandgap (2.3 eV). Tow anionic dyes, including methyl orange (MO) and acid black 1 (AB1), and two cationic dyes, such as methyl violet (MV), and rhodamine B (RhB), were applied as organic and toxic contamination in the photocatalytic examination. The process was operated under visible light for 90 min. The photodegradation reaction was carried out in three catalyst dosages of Cs_2_HgI_4_ (0.03, 0.05, and 0.07 g), indicating in Fig. 6a, 6c, and 6e, respectively. Amongst these four colorants, MO was the only colorant that exposed a degradation up to 65%. The decolorization percentage improved in all types of dyes by expanding the dosages of Cs_2_HgI_4_ as the photocatalyst. The maximum photodegradation rate was observed for methyl orange about 76.8% applying 0.07 g of Cs_2_HgI_4_. Expanding the catalyst dosages increased degradation percentage, which could be because of the enhancing the active sites and additional expansion of colorant adsorption on the surface of Cs_2_HgI_4_ [30]. Scheme 2 reveals the photocatalytic process of Cs_2_HgI_4_ over organic dyes under visible radiation. Table 2 compares the decolorization of diverse iodide nanostructures below UV or visible radiation. This table confirms that Cs_2_HgI_4_ can take part as a nano photocatalyst with other iodide mixtures. We introduce Cs_2_HgI_4_ as a novel nanocatalyst for the water disinfection operation.

**Table 2:** Comparison of photocatalytic efficiency of various iodide compounds

Sample	Highest decolorization (%)	Lowest decolorization (%)	Catalyst dosage (g)	Source of light	Ref.
Cs_2_HgI_4_	76.8 (MO)	42.8 (RhB)	0.07	Vis	This work
Rb_2_HgI_4_	72.1 (AB1)	48.1 (RhB)	0.07	Vis	[39]
Tl_4_HgI_6_/HgI_2_ nanocomposite	80.7 (RhB)	35.0 (MG*)	0.07	UV	[23]
Cu_2_CdI_4_/CuI nanocomposites	66.0 (MB**)	29.1 (MO)	0.05	UV	[40]
CsPbl_3_ nanostructures	81.7 (MV)	33.0 (AB1)	0.07	Vis	[35]
Tl_4_HgI_6_ nanostructures	76.9 (RhB)	48.9 (ThB***)	0.07	UV	[36]
TlCdI_3_ nanostructures	94.6 (MB)	27.0 (MO)	0.05	UV	[41]
Ag_2_CdI_4_ nanostructures	95.3 (RhB)	57.1 (AB1)	0.05	UV	[42]
Chitosan-coated Tl_4_PbI_6_	76.3 (ThB)	–	0.07	Vis	[43]
Tl_4_Cdl_6_ nanostructures	85.7 (AB1)	49.1 (MB)	0.05	UV	[44]

* Malachite Green

** Methylene Blue

*** Thymol Blue

The Langmuir–Hinshelwood mechanism was considered to study the reaction kinetics and to evaluate the reaction rate coefficients (k). [24]. The highest performance of photocatalytic reaction was obtained at the biggest reaction rate coefficient (Fig. 6b, 6d, and 6f). The reaction rate coefficient for MO in 0.07 g of Cs_2_HgI_4_ was calculated at k = 0.0164 min^−1^.

(6) $$\text{ln}({\text{C}}_{0}/\text{C})=\text{kt}$$

#### 3.2.1 Scavenger effect

**Fig. 7:**
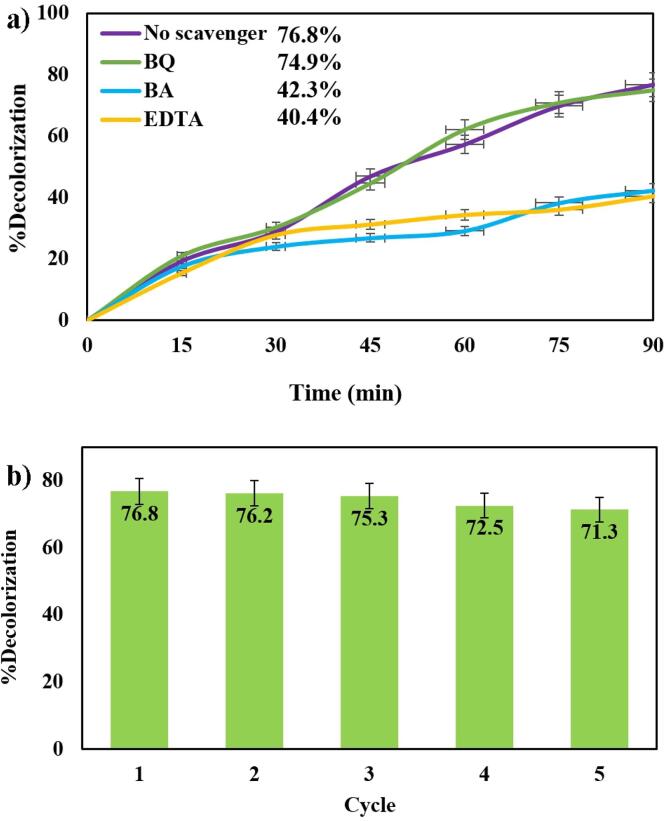
a) Photocatalytic degradation of MO with 0.07 g of Cs_2_HgI_4_ in the presence of BQ, BA, and EDTA as scavengers, b) cycling runs in the photocatalytic degradation of MO under visible irradiation.

Evaluation of the effect of active species on the degradation of dyes was carried out by a group of scavengers to study the active species. The scavengers applied in this research comprised EDTA for h^+^, BQ for O_2_•^−^, and BA for •OH [31], [32], [33]. As exhibited in Fig. 7a, the degradation performance was insignificantly diminished in the presence of BQ in comparison with no scavenger. When EDTA and BA were used as the h^+^ and •OH scavenger, an apparent decrease in photocatalytic efficiency was recognized. Concisely, the impacts of various scavengers exposed that h^+^ and •OH did an important role, and O_2_•^−^ represented an irrelevant role in the degradation of MO. Radical production is almost interconnected in the aqueous medium, and the concentration of radicals is affected by the others. The results showed that h^+^ and •OH promote the photocatalytic reaction. However, it should be noted that when other scavengers are added to the dye solution, it can reduce the production of h^+^ and •OH in the medium, thus reducing the rate of degradation.

#### 3.2.2 Photocatalytic mechanism

It is well established that the electrons of the conduction band (CB) and holes of the valence band (VB) are formed when the aqueous suspension of the catalyst is irradiated with light energy higher than its bandgap (2.3 eV) [34]. The photogenerated electrons can reduce dye or react with electron acceptors, such as dissolved O_2_ in water or adsorbed on the surface of a catalyst and reduced it to O_2_•^−^ (superoxide anion radical). The photogenerated holes can react with H_2_O or OH^–^ and oxidize them into OH• radicals or oxidize the organic dyes to produce R^+^. They have been reported to be responsible for the photodegradation of organic dyes together with other high oxidant species (peroxide radicals). Several previous studies have reported on several reactions that take place in a photocatalytic process and involve H_2_O_2_, •OH and O_2_•^−^. Hence, the relevant reactions on the Cs_2_HgI_4_ surface making the colorant degeneration can be described below [35], [36], [37], [38]:

(7) Cs_2_HgI_4_ + hν (Visible) → Cs_2_HgI_4_ (e^−^ + h^+^)

(8) Cs_2_HgI_4_ (e^−^) + O_2_ → Cs_2_HgI_4_ + O_2_•^−^

(9) Cs_2_HgI_4_ (h^+^) + H_2_O → Cs_2_HgI_4_ + H^+^ + OH•

(10) Cs_2_HgI_4_ (h^+^) + OH^−^ → Cs_2_HgI_4_ + OH•

(11) H_2_O_2_→hν2•OH

(12) H_2_O_(ads)_ + h^+^ → •OH + H^+^

(13) OH^−^_(ads)_ + h^+^ → HO•

(14) O_2_ + e^−^ → O_2_•^−^

(15) O_2(ads)_ + e^−^ + H^+^ → HO_2_•

(16) HO_2_• + HO_2_• → H_2_O_2_ + O_2_

(17) O_2_•^−^ + HO_2_• → HO_2_^−^ + O_2_

(18) HO_2_^−^ + H^+^ → H_2_O_2_

(19) H_2_O_2(ads)_ + e^−^ → •OH + OH^−^

(20) H_2_O_2_ + O_2_•^−^ → •OH + OH^−^ + O_2_

(21) H_2_O_2(ads)_ + 2 h^+^ → O_2_ + 2H^+^

(22) H_2_O_2(ads)_ + 2H^+^ + 2e^−^ → 2H_2_O

(23) H_2_O_2(ads)_ + h^+^ → HO_2_• + H^+^

(24) O_2(ads)_ + 2e^−^ + 2H^+^ → H_2_O_2(ads)_

(25) O_2_•^−^ + H^+^ ⇔ HO_2_•

(26) O_2_•^−^ + h^+^ → O_2_

(27) H_2_O_2_ + •OH → H_2_O + HO_2_•

(28) HO_2_• + •OH → H_2_O_2_ + O_2_

(29) •OH + •OH → H_2_O_2_

(30) HO_2_• + H_2_O_2_ → •OH + H_2_O + O_2_

(31) Colorant + OH• → Degradation products (e.g., CO_2_, H_2_O, H_2_)

(32) Colorant + h_VB_^+^ → Oxidation products

(33) Colorant + e_CB_^−^ → Reduction products

#### 3.2.3 Recyclability of Cs_4_HgI_4_

For checking the recyclability of Cs_4_HgI_4_ (sample 1), the catalyst was centrifuged, washed with ethanol and water, dried at 65 ℃ for 24 h, and reused five times under the same situations. As shown in Fig. 7b, Cs_4_HgI_4_ is very stable and maintains its high photocatalytic performance across five reaction cycles. Indeed during the fifth period, the reduction in photocatalytic activity is 5.5%.

## 4 Conclusions

In brief, the sonochemical route was selected to prepare Cs_2_HgI_4_ nanostructures. Several circumstances were involved in the fabrication process, such as modifying the time and power of sonication and the presence of different surfactants to produce this compound with proper particle size and morphology. The data received from SEM attested that using surfactant negatively affect the morphology and particle size. Therefore, sample 1 was preferred as the ideal sample without surfactant at the sonication power of 80 W for 20 min. Several analytical studies were conducted to assess the features of Cs_2_HgI_4_ nanostructures on the desired sample. The DRS data and bandgap estimation (2.3 eV) presented proper optical attributes of this compound in the photocatalytic procedure. The photocatalytic activity of Cs_2_HgI_4_ was operated for the degradation of cationic and anionic toxic colorants as well as several catalyst dosages (0.03, 0.05, and 0.07 g). The results showed that Cs_2_HgI_4_ could degrade anionic dyes better than cationic ones. The maximum degradation was perceived in higher catalyst dosage (0.07 g) for methyl orange (76.8%) in the visible area. Besides, the highest photodegradation efficiency was achieved at the maximum reaction rate coefficient. The reaction rate coefficient for MO in 0.07 g of Cs_2_HgI_4_ was calculated at k = 0.0164 min^−1^.

### CRediT authorship contribution statement

**Elham Abkar:** Investigation, Formal analysis, Methodology, Software. **Abbas Al-Nayili:** Writing – review & editing, Software, Visualization. **Omid Amiri:** Writing – review & editing, Software. **Mojgan Ghanbari:** Validation, Resources, Writing – review & editing, Software, Writing – original draft. **Masoud Salavati-Niasari:** Investigation, Writing – review & editing, Conceptualization, Supervision, Project administration, Validation, Resources, Visualization, Data curation, Writing – original draft.

## Declaration of Competing Interest

The authors declare that they have no known competing financial interests or personal relationships that could have appeared to influence the work reported in this paper.
